# Identification and Prevention of Antiepileptic Drug Noncompliance: The Collaborative Use of State-Supplied Pharmaceutical Data

**DOI:** 10.1155/2014/734689

**Published:** 2014-02-19

**Authors:** Joseph C. Hodges, Janet Treadwell, Amy D. Malphrus, Xuan G. Tran, Angelo P. Giardino

**Affiliations:** ^1^Baylor College of Medicine and Texas Children's Hospital, 6701 Fannin Street No. 1250, Houston, TX 77030, USA; ^2^University of Texas Southwestern Medical Center at Dallas, 5323 Harry Hines Boulevard, Dallas, TX 75390, USA; ^3^Texas Children's Health Plan, 2450 Holcombe Boulevard, Suite 34L, Houston, TX 77021, USA; ^4^Texas Children's Health Plan, Baylor College of Medicine, 2450 Holcombe Boulevard, Suite 34L, Houston, TX 77021, USA

## Abstract

*Background*. Antiepileptic drugs (AEDs) noncompliance is associated with increased risk of seizures and morbidity in seizure disorder patients. *Objective*. To identify risk factors that correlated to higher levels of morbidity, measured by emergency room (ER) utilization by seizure disorder members taking AED. *Methods*. Patients with primary or secondary diagnosis of seizures, convulsions, and/or epilepsy and prescribed AEDs during an 11-month period were included in the study. Variables were analyzed using multivariate statistical analysis including logistic regression. *Results*. The study identified 201 members. No statistical significance (NS) between age, gender, number of tablets, type of drug, or other risk factors was associated with increased mortality. Statistical significance resulted with medication compliance review of 0–14 days, 15–60 days, and 61+ days between refills. 68% of patients with ER visit had noncompliance refill between 0 and 14 days compared to 52% of patients in non-ER group (*P* = 0.04). Contrastingly, 15% of ER group had refills within 15–60 days compared with 33% of non-ER group (*P* = 0.01). There was NS difference between two groups when noncompliance was greater than 60 days (*P* = 0.66). *Conclusions*. The study suggests that careful monitoring of pharmaceutical refill information could be used to identify AED noncompliance in epileptic patients.

## 1. Introduction

Seizures are the most common pediatric neurological disorder. Of the roughly 150,000 children who experience a first-time seizure, it has been estimated that 30,000 will suffer from epilepsy [1, 2]. The terms seizure and convulsion are commonly used interchangeably, but for the purposes of this report we will simply use the term seizure [3]. Furthermore, the terminology of epilepsy and convulsive and/or seizure disorder used in this study is meant to be as inclusive as possible due to the fact that the accuracy of distinguishing an epilepsy diagnosis in the presence of seizures has been reported to range from 5% to 23% [4]. Regardless of the specific diagnosis, antiepileptic drug (AED) therapy is a hallmark therapeutic approach and the importance of adherence to AED regimes has largely been demonstrated to affect a patient's risk of future seizures [5].

Several studies have explored various risk factors and their association with AED noncompliance. Clearly, it has been shown that AED noncompliance increases a patient's risk for further seizure activity [5–9]. Furthermore poor seizure control has been linked to an increase in morbidity and mortality for patients [10, 11]. Several of these studies have explored the various risk factors predictive of AED noncompliance in order to preemptively identify the at-risk patient profile.  Table 1 lists several of the risk factors which have been previously studied and whether they were shown to increase risk of noncompliance. As shown in Table 1, it is interesting that not all previous studies agree on certain variables being correlated with an increased risk for compliance. The goal of identifying specific risk factors has been to preemptively identify the “at-risk” profile for the noncompliance patient in order to appropriately intervene in this patient population. With the lack of consensus shown between studies, it is clear that a better criterion or measure is needed to appropriately define the patient at risk of AED noncompliance.

A confounding factor of patient compliance of AEDs involves the side effect profile of a given AED [5]. A significant amount of research exists detailing the side effect profiles of various AED drugs used in the treatment of seizure disorders. Several studies have explored patient tolerance and side effect comparisons of first-generation versus second-generation AEDs [10, 12–17]. While the second-generation AEDs have shown greater tolerability and lower side effect profiles, there is an ongoing debate about the cost effectiveness of these newer AEDs [12]. Furthermore, discomfort on the part of general pediatricians (GPs) in prescribing second generation AEDs has been shown to cause large variations in the use of second-generation AEDs by GPs [18]. A continued interest in the long-term effect of AEDs in patients also adds to the discomfort of GPs when managing seizure patients in the primary care setting [19]. Due to the general levels of discomfort of GPs in prescribing these AEDs, the resulting shift in care from general pediatricians to pediatric neurologists further contributes to an existing problem of a lack of access to pediatric neurologists [20].

Regardless of the generation of drug used by the patient with seizures, the side effect profiles still represent a considerable burden on patients [15, 16]. This has led to a focus on managing not only seizure activity but also quality of life as it is affected by the side effect burden of antiepileptics [17]. It is important to recognize that patient noncompliance has previously been shown to be correlated to the side effect burden of the prescribed AEDs [5].

In addition to the side effect profile of a given AED, the complex and variable pharmacodynamics and pharmacokinetics of AEDs are also confounding factors of noncompliance and risk for further seizures. Historically, therapeutic drug monitoring has been the gold standard in identifying as well as verifying the noncompliant patients [21]. The approach has been criticized as being both expensive and invasive. As stated previously, in order to provide an alternative to invasive therapeutic drug monitoring (TDM) and to preemptively identify noncompliance, researchers have focused on risk factors of noncompliance. All previous studies as shown in Table 1 have used TDM to verify noncompliance. Despite early reports of the usefulness of TDM [22], several more recent studies have shown that TDM does not affect patient outcome [23–25] which largely justifies more recent attempts to identify noninvasive clinical, social, and demographic risk factors as more appropriate tools for identifying noncompliance [23–26]. The ultimate goal of such risk factor studies has been to define standardized criteria by which to identify AED noncompliance.

However, Snodgrass and Parks [26] explained that one advantage to TDM is that its use can identify noncompliance as well as pharmacokinetic variability. This variation in the pharmacokinetics captured by TDM has been used to justify higher dosages in such patients [21, 22, 26, 27]. With respect to a noncompliant patient, this dose escalation is a potentially harmful practice as failure to verify and distinguish a noncompliant patient versus a patient with variable pharmacokinetics can lead to the overtreatment of noncompliant patients [8]. In addition to this complex scenario, it is important to realize that the variable of pharmacodynamics, or the actual patient responsiveness, is not captured by TDM and therefore provides another counter argument against its clinical usefulness [8, 28]. In other words, some patients may remain clinically stable despite subtherapeutic serum levels of the AED simply because of variations in pharmacodynamics among patients.

The current consensus on TDM is that each patient should be treated on an individual basis as disease severity and drug response are highly variable [29, 30]. The Scottish Intercollegiate Guidelines Network (SIGN) number 70 [31] states that the use of TDM should not be routine. The guideline does indicate that in the scenarios of AED adjustments and toxicity assessments TDM can be useful [31]. It is clear that in the appropriate management of AEDs, the entire clinical scenario must be taken into account with TDM being only one aspect of a very thorough clinical evaluation [25]. Despite these guidelines, there is evidence that the use of TDM is still inappropriately overutilized [28]. In the absence of a standardized evidence-based risk factor guideline, it should be expected that TDM will continue to be overutilized as an invasive and costly method for identifying AED noncompliance [28]. However, in the absence of a consensus on previously studied risk factors and the absence of clinically established and useful criteria, we attempt to define an alternative collaborative approach between health plans and health providers through the judicial use of state-supplied pharmaceutical administrative claims data.

At the time of the study, our HMO administers Medicaid (STAR) and State-Children's Health Insurance Program (S-CHIP) to over 160,000 pediatric members. In light of previous studies, we have a patient population which includes several well-described clinical, social, and demographic risk factors such as illness burden, presence of side effects, income level, race, and intellectual level. Given the limited resources to better manage what appears to be a large number of at-risk members, our plan attempted to identify who is at the highest risk within our plan. Through the use of our vast resource of claims data and the findings of past research, we sought to identify practical criteria for applying risk profiles to our patients who take AEDs. We believed that identifying variables correlated to a higher morbidity risk would help improve the quality of care of our patients who suffer from seizure disorders. Furthermore, given the current crisis of access to pediatric neurologists in our local areas of coverage, we have noticed a large increase in ER and inpatient utilization which we defined as evidence of both increased morbidity and decreasing patient access to specialists [20, 32, 33]. While limited access may impact the increased use of ER and inpatient utilization, we sought to identify other aspects which may contribute to the increased morbidity we have identified among our patients. The ultimate purpose of the study was to identify the patients who are at risk for increased seizure activity and thus higher disease morbidity in order to more effectively use health plan resources and physician management to improve the quality of healthcare for these patients.

## 2. Methods

### 2.1. Study Design

Using existing HMO claims data for office visits, ER, and inpatient stays (IP) utilization as well as state-supplied pharmaceutical data, we constructed a database of all patients with the diagnosis of convulsions of any type and epilepsy of any type. All patients had three or more months of AED pharmaceutical refill history. We selected all plan members who were seen in the office based setting by the collaborating pediatric neurology practice for convulsions and epilepsy as either a primary, secondary, or tertiary diagnosis. We then constructed a database using ICD-9 codes (345.xx or 780.xx) for convulsions and epilepsy of any type to capture ER and IP utilization (Table 2). This list was checked against whether or not the patient had an office visit with the collaborating pediatric neurology practice to ensure the validity of the two databases. Of the 649 patients seen for any reason by the pediatric neurology practice, 291 were seen for convulsions or epilepsy (345.xx or 780.xx).

The next step in building the model was to incorporate state pharmaceutical data for AEDs. To accomplish this we constructed a state pharmaceutical database of the 291 convulsion and/or epilepsy (C/E) group. Ninety patients met the exclusion criteria of not having either ER, office-based, or pharmaceutical data. This exclusion group represents a limitation to the study as it is highly possible that this group included noncompliant patients. However, without the exclusion of this group, the model would have significantly limited the number of variables used in the multivariate analysis due to the lack of certain claims data for these patients.

### 2.2. Statistical Analysis

After the exclusion of 90 patients from the original 291 patient cohort, the model constructed a patient list of 201-patients who had an office visit, ER, and pharmacy utilization data. We used this 201 patient list to perform the multivariate analysis using logistic regression. The dependent variable was the utilization of ER services, which we defined as the measure of morbidity associated with these patients [33]. The analysis explored the independent variables shown in Table 3 and their relationship with the dependent variable of morbidity. A *P* value of less than or equal to 0.05 was considered statistically significant. Statistical analysis was calculated using SPSS (version 15.0). Institutional Review Board approval was obtained and all data was collected under the regulations of the Health Insurance Portability and Accountability Act in regard to patient information privacy.

### 2.3. Limitations of Design

While the purpose of the study aimed at using only existing claims data in order to find practical and easy markers for noncompliance, we acknowledge that our study was also limited by this design. By limiting the methods of this study to include only existing claims data, we were unable to capture many of the frequently studied risk factors for noncompliance such as intellectual level, involvement of caretaker, duration of disease, experience of side effects, office visit followup information, illness burden, and physician-patient communication. Much of this information would have required a chart review, which was not performed on the 201-patient subgroup. Despite this limitation, we believe this study's unique approach of using widely available and noninvasive claims data justifies this study limitation as such approach has a substantial potential to decrease the morbidity associated with AED noncompliance.

## 3. Results

### 3.1. Demographics and Descriptive Statistics

There were 121 males and 90 females in the final list of 201 patients. The average age of the group was 6 years. Of these 201 patients, the largest subgroup included 149 (68%) of patients who were effectively managed in an office-only setting. This group was designated the “non-ER” group for office-only management. Fifty-nine (27%) patients presented to the ER during the 11-month period. This group was designated the “ER” group for emergency room utilization. It is interesting that in terms of all patients in our HMO who were followed by the collaborating pediatric neurology group this C/E group captured 41% of all neurological ER presentations.

### 3.2. Results of Multivariate Analysis

As previously discussed, the dependent variable was designed to capture a measurement of morbidity by categorizing a patient based on ER utilization.  Table 4 describes the independent variable and whether their respective correlations were shown to be significantly correlated to the dependent variable of morbidity as measured against ER utilization.

**Figure 1:**
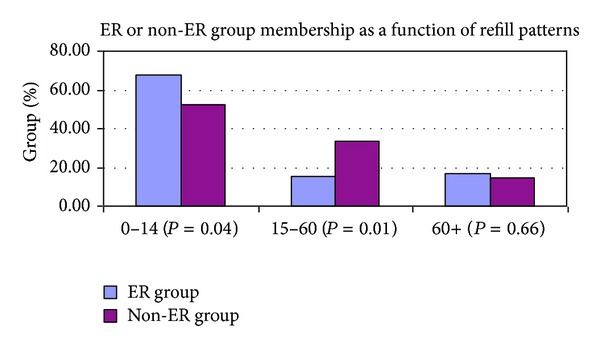
Various risk factors for AED noncompliance.

The results showed that available data of prescription refills were the most useful in predicting morbidity of patient with the diagnosis of convulsion and/or epilepsy. Historically, noncompliance has been evaluated and measured by voluntary patient reporting or therapeutic drug monitoring. These approaches are often not readily available or invasive and expensive, respectively. It is interesting that our study showed that a failure to refill an AED prescription during the first two weeks (14 days) was positively correlated with ER or IP utilization.  Figure 1 shows that when comparing the group who presented to the ER (ER group) and the group who was effectively managed on an ambulatory-only basis (non-ER group), the ER and non-ER groups were significantly different in the 0–14-day group and in the 15–60-day group. The groups were not statistically different in the 61+ day group. All other variables under analysis did not show a statistically significant correlation to morbidity. Medicaid or S-CHIP did show co-linearity due to the fact that our plan administers 100% of its plans for these two types of plans.

### 3.3. The Non-ER Group

**Figure 2:**
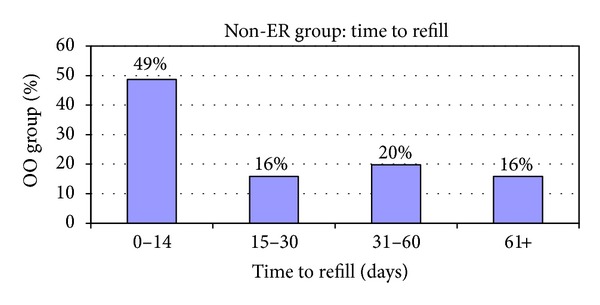
Frequent ICD-9 codes for the initial cohort construction.

The exclusively office-based managed group represented 142 patients (74%) of the entire C/E study group. The breakdown of these 142 patients in terms of refill patterns is shown in Figure 2. Seventy-three (49%) of the non-ER group patients would be considered at risk for increased morbidity based on the fact that they show evidence of refill delays of during the first two weeks.

### 3.4. The Emergency Room (ER) Group

**Figure 3:**
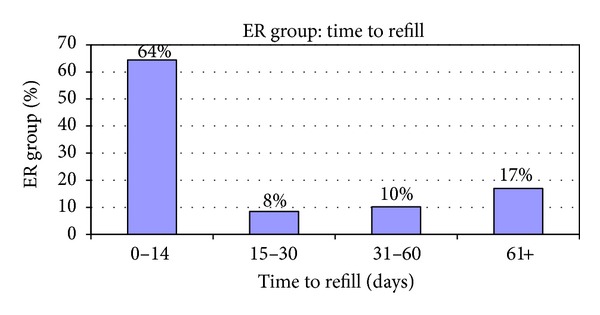
Independent variables used in the model.

The emergency room group represented 59 patients (26%) of the entire C/E study group. The breakdown of this ER group showed that 18 members accounted for 44 of the 86 ER visits (51%). We further analyzed these frequent utilizers through a general chart review. Further analysis of this group revealed that 6 of these 18 frequent utilizing patients presented for a new diagnosis for convulsion or epilepsy as they had no previous office-based management and were placed on AEDs after this ER presentation. Three of these 6 patients revisited the ER two weeks after their initial presentation to the ER despite having followup with the pediatric neurology group within 2 weeks of initial ER presentation. Also, 7 patients were previously diagnosed and seen by the pediatric neurology group but showed pharmaceutical evidence of noncompliance as well as heavy ER or IP utilization. The remaining 5 of the 18 frequent ER utilizers showed refill compliance and preexisting disease.  Figure 3 summarizes these findings. Despite these intricacies to this group, the results showed that 33 patients (64%) visited the ER with two weeks after a missed prescription refill.

### 3.5. The Inpatient (IP) Group

**Figure 4:**
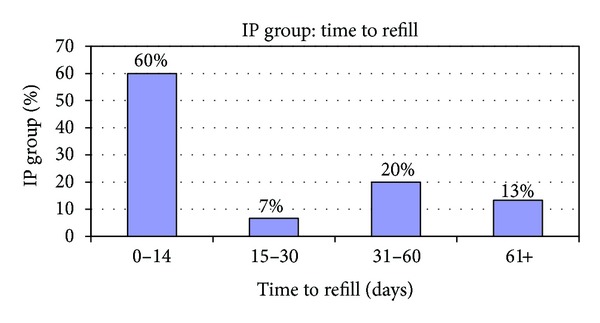
Results of multivariate analysis.

**Figure 5:**
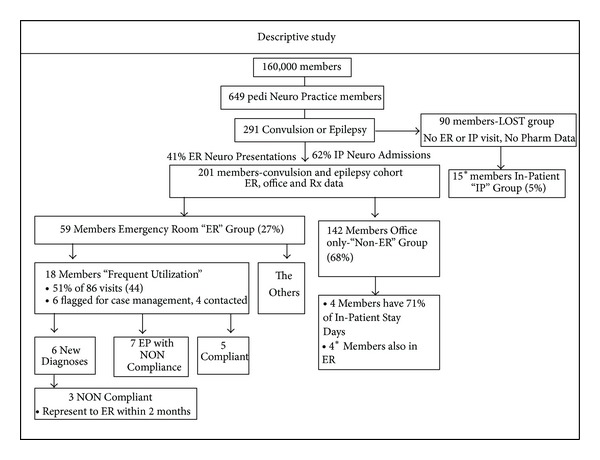
Summary of Descriptive Study.

While we did not include the in-patient (IP) group in the study due the complexity levels associated with these patients, this IP group represented only 15 patients of all the patients with convulsions or epilepsy in the health plan. The breakdown of this IP group showed that four members accounted for eight of the 13 IP stay days visits (71%). We further analyzed these frequent utilizers through a general chart review. This review showed that four of the 15 patients were also captured as ER patients as they were admitted after an ER presentation. The rest of the admissions were either direct admits from clinic or transfers. Again, it is interesting that the majority of the patients who were admitted due to high morbidity associated with their seizure were part of the 0–14-day refill pattern group.  Figure 4 summarizes these findings. In addition, all descriptive findings are summarized in Figure 5.

## 4. Discussion

The findings of this study highlight three important aspects surrounding healthcare management and pediatric subspecialties. First, existing HMO claims data, specifically pharmaceutical refill adherence, can be easily used to help describe and identify at-risk chronic disease populations. Secondly, it is clear that the effective use of such findings will require open communication between healthcare insurance companies and physicians in order to improve the quality of patient care. Lastly, the effective management of such chronic care at-risk populations will require adequate access to care which is a specific problem in pediatrics due to the low supply of pediatric subspecialists [20, 32, 33].

### 4.1. Refill Compliance

Refill compliance (RC) using pharmaceutical data from administrative claims data has been studied as a surrogate to using other clinical, demographic, and social risk factors for pharmaceutical compliance [34, 35]. It has been demonstrated that RC can be documented in as many as three different measures: (1) availability of drug as measured by a ratio of time, (2) availability of a drug as measured at a certain fixed time, (3) or the time between refills [36]. Our study used the latter of the three and this measure has commonly been referred to a medication gap [37]. It has previously been shown that the time between refills is the easiest and most useful measurement to determine RC [36].

While the use of RC has been shown to be valuable in identifying noncompliance, the use of such data is not currently widespread in the healthcare system [38, 39]. The use of such information has been shown to be cost effective, noninvasive, and easy to use in several chronic care situations [37, 39, 40]. Given the proven usefulness of measuring adherence through the use of pharmacy administrative databases, several efforts have been made to standardize measurement methods, terminology, and definitions of refill compliance [41]. Furthermore, Bryson et al. reported an algorithm to be used over short observational periods to help standardize the use of refill data [37]. The results of this study showed improvement against those of other existing refill algorithms with respect to the very specific scenario of the treatment of hypercholesterolemia. More research is justified to help characterize the usefulness of existing RC algorithms to the vast number of clinical scenarios in which it is potentially useful. While standardized algorithms may help increase the adoption of refill data as a useful clinical tool, it is clear that chronic disease scenarios must be considered individually. Disease-specific algorithms which consider unique patient populations, chronic disease scenarios, and specific pharmaceuticals will mostly likely be necessary in order to identify how best to utilize pharmaceutical refill data [17].

### 4.2. A Special Consideration: Withdrawal of AEDs

Our findings support the use of widely available pharmaceutical refill data to identify noncompliance in patients taking AEDs. However, the use of such data will naturally capture patients who are either self-withdrawing or withdrawing under guidance of a physician. Our data captured a small percentage of patients who historically showed compliance but recently showed evidence of noncompliance. It is possible that this subset of patients represented a withdrawal group. The presence of such a group within the at-risk group described in this study again highlights the importance of collaboration between physicians and healthcare plans in identifying these patients. While studies have yet to prove whether rapid (less than 6 weeks) or slow (greater than 3 months) withdrawal leads to greater disease-free timeframes, it is clear that the close management of withdrawing patients improves the success of AED withdrawal [31, 42–44]. While the importance of treating physicians involvement is highly valuable, the ability of healthcare plans to use case management to help comanage these withdrawing patients could help facilitate successful AED withdrawal [45]. Due to the frequent changes in dosages during withdrawal the potential impact of case management could be significant in helping educate and monitor the withdrawal process.

## 5. Conclusion

Our study's finding which correlates to refill adherence and morbidity in patients taking antiepileptic drugs highlights the ease and cost effectiveness of using existing HMO claims data to identify at risk populations. Furthermore, the strength of the positive correlations which range from 0.1892 to 0.1524 suggests that refill information may be one of the most valuable variables yet described in helping to identify noncompliant patients. This correlation in predicting an increase in mortality would benefit from a prospective study in order to verify its clinical usefulness. Due to the fact that several of the risk factors previously studied have not consistently been shown to be correlated to noncompliance, we believe the strength of our findings further validates its potential clinical usefulness.

Should such HMO data be used to identify at-risk patients, we acknowledge that communication and collaboration between health plans and physicians must be strengthened in order to translate data findings into improvements in clinical healthcare quality. In addition, due to the frequency of AED adjustments, it is clear that the impact of such quality improvement efforts will require adequate access to office-based management for at-risk patients as well as intense case management by HMO plans. This consideration is extremely important in light of the nationally demonstrated lack of access to pediatric neurologists.

Ultimately, our findings support three current themes in quality improvement initiatives as they apply to the specific situation of AED noncompliance: (1) the identification of “high-risk” or “at-risk” patient populations through the easy use of administrative refill data to identify at-risk noncompliant patients, (2) improved communication and collaboration between health plans and physicians in order to effectively utilize this data, (3) the importance of access to adequate care due to the often complicated management of AEDs.

It is our hope that the findings of this report will encourage healthcare plans and physicians to collaboratively use readily available administrative claims data to help identify chronic care populations at risk for increased morbidity due to noncompliance. The success of such quality improvement initiatives often hinges on the collaborative efforts between the numerous parties involved in the complex environment of healthcare delivery. More research describing such collaboration between health plans and physicians should be undertaken as the opportunities for such efforts are vastly underrecognized.

## Figures and Tables

**Table 1:** Various risk factors for AED noncompliance.

Risk factor	Study	Increased risk	Noncompliance marker
Type of seizures	Al Faris, Santiago, Snodgrass, Loiseau	No	TDM
Illness burden	Santiago, Snodgrass, **Loiseau**	Yes	TDM
Physician-patient communication	**Santiago**	Yes	TDM
Total number of pills per day	**Santiago, Loiseau**	Yes	TDM
Age	Santiago, **Buck, Snodgrass, **Loiseau	Yes	TDM
Gender	Santiago, Loiseau	No	TDM
Intellectual level	**Santiago**	Yes	TDM
Compliance with appointment/followup	**AlFaris, Snodgrass**	Yes	TDM
Perceived stigma	**Buck**	Yes	TDM
Experience of side effects	**Buck**	Yes	TDM
Race	**Snodgrass**	Yes	TDM
Reliance on social security	**Snodgrass**	Yes	TDM
Insured status	**Snodgrass**	Yes	TDM
Involvement of caretaker/parent	**Snodgrass**	Yes	TDM
Type of AED	**Al Faris, Lusic**	Yes	TDM
Duration of disease	**Loiseau**	Yes	TDM

Bold: study that found correlation.

Unbold: study that explored relationship but did not find a correlation.

TDM: therapeutic drug monitoring.

**Table 2:** Frequent ICD-9 codes for the initial cohort construction.

ICD code	Description
780.36	Other convulsions
780.39	Convulsions NEC
345.1	Generalized convulsive epilepsy without intractable epilepsy
345.3	Grand mal status epileptic
345.9	Epilepsy unspecified without intractable epilepsy
345.5	Partial epilepsy without impairment of consciousness without intractable epilepsy
345.4	Psychomotor epilepsy without intractable epilepsy
345.6	Infantile spasm without intractable epilepsy
345.71	Epilepsia partialis continua without intractable epilepsy
345.9	Epilepsy, unspecified numbers without intractable epilepsy

**Table 3:** Independent variables used in the model.

Independent variable	Description
1–14 days refill history	Patient showed at least one refill history delay of 1–14 days
15–30 days refill history	Patient showed at least one refill history delay of 15–30 days
31–60 days refill history	Patient showed at least one refill history delay of 31–60 days
61+ days refill history	Patient showed at least one refill history delay of 61+ days
Medicaid/S-CHIP	Patients insurance was Medicaid or SCHIP
Age	Number of years of life
Gender	Sex of child
Number of pills (per day)	Number of AED pills to be taken in a 24-hour period
Type of seizure	Based on ICD-9 code
Type of AED	All AED included in model
Race	As reported to health plan
Member months	Number of months of enrollment in HMO plan

**Table 4:** Results of multivariate analysis.

Independent variable	Significance
**0–14 days refill history**	**Yes (*P* = 0.04) Positive correlation **
**15–30 days refill history**	**Yes (*P* = 0.001) Negative correlation**
61+ days refill history	No (*P* = 0.66)
Medicaid/S-CHIP	Colinearity
Age	No
Gender	No
Number of pills per day	No
Type of seizure	No
Type of AED	No
Race	No
Member months	No

Bold font shows the Statically significance.
